# Multimorbidity and Its Patterns according to Immigrant Origin. A Nationwide Register-Based Study in Norway

**DOI:** 10.1371/journal.pone.0145233

**Published:** 2015-12-18

**Authors:** Esperanza Diaz, Beatriz Poblador-Pou, Luis-Andrés Gimeno-Feliu, Amaia Calderón-Larrañaga, Bernadette N. Kumar, Alexandra Prados-Torres

**Affiliations:** 1 Department of Global Public Health and Primary Care, University of Bergen, Bergen, Norway; 2 Norwegian Centre for Minority Health Research, Oslo, Norway; 3 EpiChron Research Group on Chronic Diseases, Aragón Health Sciences Institute (IACS), IIS Aragón, Miguel Servet University Hospital, Zaragoza, Spain; 4 Red de Investigación en Servicios de Salud en Enfermedades Crónicas (REDISSEC), Carlos III Health Institute, Madrid, Spain; 5 San Pablo Health Centre, Zaragoza, Spain; 6 University of Zaragoza, Zaragoza, Spain; 7 Institute for Health and Society, University of Oslo, Oslo, Norway; Mayo Clinic, UNITED STATES

## Abstract

**Introduction:**

As the flows of immigrant populations increase worldwide, their heterogeneity becomes apparent with respect to the differences in the prevalence of chronic physical and mental disease. Multimorbidity provides a new framework in understanding chronic diseases holistically as the consequence of environmental, social, and personal risks that contribute to increased vulnerability to a wide variety of illnesses. There is a lack of studies on multimorbidity among immigrants compared to native-born populations.

**Methodology:**

This nationwide multi-register study in Norway enabled us i) to study the associations between multimorbidity and immigrant origin, accounting for other known risk factors for multimorbidity such as gender, age and socioeconomic levels using logistic regression analyses, and ii) to identify patterns of multimorbidity in Norway for immigrants and Norwegian-born by means of exploratory factor analysis technique.

**Results:**

Multimorbidity rates were lower for immigrants compared to Norwegian-born individuals, with unadjusted odds ratios (OR) and 95% confidence intervals 0.38 (0.37–0.39) for Eastern Europe, 0.58 (0.57–0.59) for Asia, Africa and Latin America, and 0.67 (0.66–0.68) for Western Europe and North America. Results remained significant after adjusting for socioeconomic factors. Similar multimorbidity disease patterns were observed among Norwegian-born and immigrants, in particular between Norwegian-born and those from Western European and North American countries. However, the complexity of patterns that emerged for the other immigrant groups was greater. Despite differences observed in the development of patterns with age, such as ischemic heart disease among immigrant women, we were unable to detect the systematic development of the multimorbidity patterns among immigrants at younger ages.

**Conclusions:**

Our study confirms that migrants have lower multimorbidity levels compared to Norwegian-born. The greater complexity of multimorbidity patterns for some immigrant groups requires further investigation. Health care policies and practice will require a holistic approach for specific population groups in order to meet their health needs and to curb and prevent diseases.

## Introduction

Multimorbidity is highly prevalent among older people, women and those with lower socioeconomic levels, but not exclusive to these groups [1–3]. Patients with multimorbidity often present with lower function levels, higher levels of polypharmacy, poorer quality of life, increased health care utilization and mortality rates over and above the risk attributable to individual diseases [2, 4–7].

Multimorbidity provides a holistic framework in understanding chronic diseases as the consequence of environmental, social, and personal risks that contribute to increased vulnerability to a wide variety of illnesses as opposed to studying mental and physical health diseases one by one [8–10]. Several studies have attempted to disentangle the different multimorbidity patterns; i.e. the non-random positive association of specific diseases and health problems, also called associative multimorbidity [9]. Despite the varying populations and methodologies, three common patterns of multimorbidity are observed in these studies: cardiovascular-metabolic, mental health and musculoskeletal [2, 9, 11]. In total up to one hundred different multimorbidity patterns have been identified [9].

The proportion of immigrants is growing in Europe [12], but they are heterogeneous, both in their origins, status and migration histories. Many different theories can contribute to hypothesise an association between migration and multimorbidity. According to the “healthy immigrant theory”, some immigrants are healthier than the host population of their new country because they represent a selected and healthier subgroup of their country of origin population [13]. On the other hand, even in the presence of better health at arrival, the health of immigrants worsens quicker than non-immigrants after arrival [14, 15]. Although the theories related to migration are still evolving, migration itself can increase vulnerability to environmental, social, behavioural and psychological risks [12] during and/or after the migration process for all immigrants [16–19]. However, the healthy immigrant theory may not apply to refugees and asylum seekers, who are forced into migration and therefore have generally poorer mental and physical health compared to the host population [20]. A study on multimorbidity among young asylum seekers in Switzerland showed a relatively high prevalence of multimorbidity [16], thus concurring with this theory. Also, in a recently published study of immigrants in Norway [21], multimorbidity was significantly lower among labour and education immigrants, but higher among refugees, compared to family reunification immigrants.

Compared to the Norwegian-borns, a lower percentage of immigrants use primary care services but once they are in contact with health care, they often become frequent users [22, 23]. Although significant differences regarding use of services for both psychological [24] and physical [22] diagnoses for immigrants and Norwegian- borns have been observed, none of the published studies have assessed the patterns of the global burden of disease, leaving the health picture of immigrants rather fragmented. Two recent international reviews have highlighted the scarcity of knowledge about multimorbidity for patients from lower and middle-income countries [1], and for immigrants [25].

Beside the effects of the reasons for migration on health, country of origin of the migrants must also be taken into consideration due the global variance in the prevalence of specific diseases, in addition to interactions between genetic and migration factors [26]. Particularly relevant are the high prevalence of diabetes mellitus among immigrants from South Asian and some African countries [27, 28] or of cardiovascular diseases among immigrants from South Asians and Eastern countries [29]. However, to the best of our knowledge, no study to date has studied multimorbidity and its patterns among immigrants from different geographical regions compared to a native-born population.

This nationwide multi-register study in Norway enabled us i) to study the associations between multimorbidity and immigrant status as classified by area of origin, accounting for other known risk factors for multimorbidity, and ii) to identify patterns of multimorbidity in Norway for immigrants and Norwegian-born at different ages. Based on the previously described existing theories and on our earlier studies on use of health care services in Norway [22, 23, 30], our hypothesis was that immigrant groups would have lower rates of multimorbidity compared to Norwegian-born. Associations between length of stay in Norway and use of health services in our previous studies indicate that health worsens quicker for migrants and therefore we hypothesize that they develop multimorbidity patterns at a younger age.

## Methods

This register-based study relies on merged data from the National Population Register and the Norwegian Health Economics Administration database (HELFO). The personal identification number assigned to Norwegian citizens and to legal immigrants staying in Norway for at least six months was used to link the registries. Irregular immigrants without legal residence and regular migrants staying for shorter periods were not included in the study.

All 15 year old or older Norwegians (n = 3,349,721), defined as born in Norway with both parents from Norway, and immigrants (n = 389,807), defined as born abroad with both parents from abroad, registered in Norway in 2008 were included in the study. Other categories like born in Norway with one or both parents from abroad and adopted children were excluded because of their low numbers among immigrants older than 15 years old. Information on gender, age, personal income level and country of origin was obtained from the National Population Register for all study subjects. Age was categorized into three groups: 15–44, 45–64 and 65+ years. Income level was categorized in four levels: low (under 50,000 Norwegian Crowns (NOK)), medium (50,001 to 200,000 NOK), high (200,001 to 400,000 NOK) and very high (over 400,000 NOK). According to Statistics Norway, countries of origin were classified into six broad areas: North America and Oceania, Nordic countries, West Europe excluding Turkey, Eastern Europe and Africa, Asia including Turkey and Latin America together [31]. We conducted analyses for each of these regions but, for the sake of parsimony and in order to have enough persons in all age categories, we recoded areas with similar characteristics into North America and Western Europe excluding Turkey (named as “Western countries”, n = 109,428), Eastern Europe (n = 99,301) and Africa, Asia including Turkey and Latin America together (named as “Other non-Western countries”, n = 181,068). A list with the major countries represented in each of the areas is presented as supplemental material in S1 Table.

The HELFO-database contains claims for all patient contacts within the public primary health care services including both consultations with general practitioners (GPs) and emergency room (ER) services. Each claim contains at least one medical diagnosis based on the International Classification of Primary Care (ICPC-2) registered by the physician. These ICPC-2 diagnoses originally sampled in 2008 for reimbursement and administrative purposes were grouped according to the Expanded Diagnostic Clusters (EDC) of the Johns Hopkins University Adjusted Clinical Groups (ACG^®^) Case-Mix System [5]. The EDC methodology assigns ICPC-2 codes found in claims to one of 269 EDCs. As broad groupings of diagnosis codes, EDCs help to remove differences in coding behaviour between practitioners. The 114 chronic EDCs included in the study were selected based on the list published by Salisbury et al in 2011 [32].

### Analyses

Descriptive analyses were conducted. Morbidity level was presented as the proportion of patients with none, one, or two or more chronic EDCs registered during the year 2008. A dichotomous multimorbidity variable based on the total number of the selected EDCs registered for each person was created, defined by two or more different chronic diagnoses [10]. For this dependent variable, binary logistic regression analyses were conducted in four steps. Firstly, the independent variables age, gender, immigrant area of origin and income level were included one at the time. Secondly, the first three variables were included together in Model 1. Thirdly, all four independent variables were included simultaneously in Model 2. Lastly, Model 3 also included the number of visits to primary care in 2008. Analyses were also conducted with the multimorbidity variable being three or more chronic EDCs, obtaining similar results, not shown in the article.

To determine multimorbidity patterns, an exploratory factor analysis technique was applied by gender and age category, and for Norwegian-borns and the three defined immigrant groups separately. This methodology has been thoroughly described by Prados-Torres et al [33], and includes only EDCs with prevalence equal to or greater than 1% for each age and gender subgroup studied. Due to the dichotomous nature of the EDC variables, tetra-choric correlation matrices were conducted to determine which EDCs were included in each factor, with no restriction regarding the number of patterns in which each EDC could be included. The factors resulting from these matrices were interpreted as multimorbidity patterns (chronic EDCs related to each other), scoring between -1 and 1 depending on the strength of the association of each of the EDCs to the disease pattern. To determine the number of factors to extract, a scree plot representing the eigenvalues of the correlation matrix in descending order was utilized, extracting the number of factors that corresponded to the sequence number of the eigenvalue that produced the inflection point of the curve. When a clear solution was not obtained by this method, a clinical approach based on the authors’ expertise was used to determine the patterns with the most plausible pathophysiologic explanation. The same strategy was used when factor scores were greater than 1 (Heywood phenomenon). An oblique rotation (Oblimin) was applied, allowing the factors to be correlated with one another, and EDCs with scores equal to or greater than 0.25 for each factor were selected for the relevant multimorbidity patterns. The adequacy of the sample used to perform the factor analysis was measured using the Kaiser-Meyer-Olkin (KMO). This parameter takes values between 0 and 1, which are closer to 1 with a greater goodness of fit. Analyses were conducted in SPSS 20.0 and Stata 13.0.

This study is part of the project “Immigrants’ health in Norway”, which was approved by the Regional Committee for Medical and Health Research Ethics, the Norwegian Data Inspectorate, the Norwegian Labour Welfare Service and the Norwegian Directorate of Health. The Norwegian Social Science Data Service prepared the final data file.

## Results

**Fig 1:**
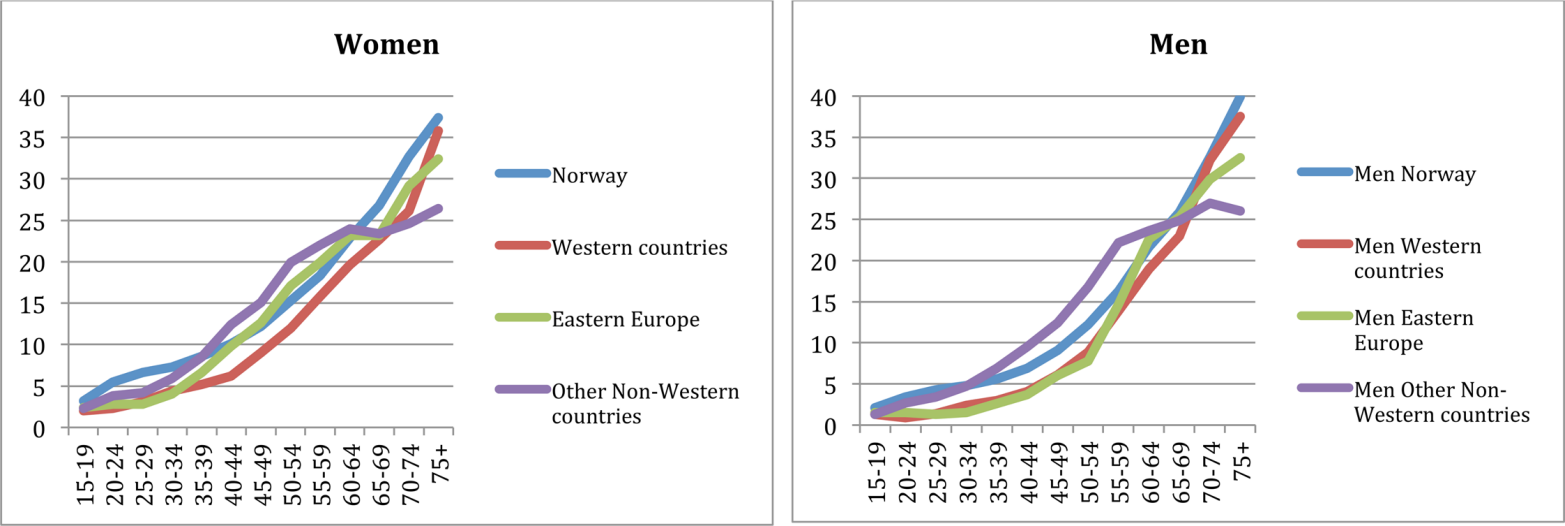
Multimorbidity by age. Norwegian-born and immigrants by gender.

Demographic characteristics of Norwegians and immigrants are presented in Table 1. With few exceptions, women were underrepresented across immigrant groups. While immigrants from Western countries had higher income levels than Norwegians, income levels were lower for the rest of immigrants. Norwegians had more chronic conditions registered compared to immigrants except for those younger than 65 years from other non-Western countries. Fig 1 depicts the proportion of Norwegians and immigrants with multimorbidity by age and gender. Male and female 30 to 60 years old immigrants from other non-Western countries showed higher multimorbidity compared to Norwegians of the same age, but this was the group of origin with lowest global prevalence of multimorbidity among the older age groups.

**Table 1:** Demographic and health information for natives and immigrants in Norway.

	Norwegian-born			Western Countries (West Europe & N. America)			Eastern Europe			Other Non-Western (Asia, Africa & Latin America)		
Age (years)	15–44	45–64	65+	15–44	45–64	65+	15–44	45–64	65+	15–44	45–64	65+
Numbers	1,557,485	1,086,136	706,100	56,564	35,508	17,366	73,425	21,957	3,919	131,929	42,848	6,291
Women, %	48.8	49.5	56.7	45.8	44.9	62.5	43.4	43.5	56.0	53.2	46.5	50.9
Income level (in 1000 NOK per year), %												
Low (<50)	23.6	20.9	89.8	19.5	20.8	86.2	25.3	27.6	91.3	41.2	45.0	92.4
Medium (50–200)	17.3	10.0	5.2	16.3	10.8	6.1	23.4	15.1	4.0	19.9	11.9	3.3
High (200–400)	32.2	32.1	2.8	34.1	29.4	3.8	39.5	39.8	2.5	27.1	26.5	2.7
Very high (>400)	26.9	37.0	2.1	30.1	39.0	3.9	11.8	17.5	2.2	11.7	16.6	1.6
Number of chronic conditions registered in 2008, %												
None	76.4	56.3	33.0	83.6	64.3	38.1	86.1	68.3	40.7	76.4	54.8	44.1
One	17.8	27.7	33.0	13.0	23.3	31.7	10.7	19.2	30.5	17.7	27.5	30.7
Two or more	5.8	16.0	34.0	3.4	12.4	30.2	3.2	12.5	28.8	5.9	17.6	25.2
Number of visits to GP or ER in 2008												
Mean (SD)	2.4(3.5)	3.0(3.9)	4.2 (4.8)	1.8(2.9)	2.5(3.5)	3.9(4.7)	1.7(3.1)	2.6 (3.9)	3.4 (4.3)	2.9(3.9)	3.9(4.6)	3.5(4.4)

The logistic regression analyses performed showed that multimorbidity was significantly associated with female gender, with a clear dose-response association for age and income level in all models. Immigrants had a significantly lower probability of multimorbidity compared to Norwegian-borns, with Eastern Europeans showing the lowest odds. Although the probability of multimorbidity was significantly higher for immigrants from other non-Western countries when adjusting for age and gender, this difference disappeared when adjusting for income level. Also the associations between multimorbidity, age and gender were moderated by the inclusion of income level in the model (Table 2). To study potential interactions, analyses were conducted separately by gender, showing associations in the same direction, although men from Eastern European countries had even lower adjusted odds ratio (OR) for multimorbidity (0.49, 95% CI 0.47–0.51) compared to women from the same origin (0.70, 95% CI 0.67–0.73) in Model 2. The number of visits to primary health care included in Model 3 increased the goodness of fit of the model, but it did not change the direction of the results.

**Table 2:** Associations between multimorbidity and immigrant status. Binary logistic regression analyses.

	Unadjusted		Adjusted Model 1		Adjusted Model 2		Adjusted Model 3	
	*OR*	*95% CI*	*OR*	*95% CI*	*OR*	*95% CI*	*OR*	*95% CI*
*Age in years*								
15–44 (ref)	1		1		1		1	
45–64	3.17	3.14–3.19	3.14	3.12–3.17	3.44	3.41–3.47	3.21	3.18–3.24
65+	8.57	8.50–8.64	8.37	8.30–8.44	5.14	5.10–5.19	4.58	4.53–4.63
*Gender*								
Men (ref)	1		1		1		1	
Women	1.29	1.29–1.30	1.19	1.18–1.20	1.06	1.05–1.06	0.90	0.89–0.90
*Immigrant area of origin*								
Norwegian-born (ref)	1		1		1		1	
Western Europe & North America	0.67	0.66–0.68	0.74	0.72–0.75	0.75	0.74–0.77	0.81	0.79–0.83
Eastern Europe	0.38	0.37–0.39	0.65	0.64–0.67	0.59	0.57–0.61	0.64	0.63–0.66
Asia, Africa & Latin America	0.58	0.57–0.59	1.02	1.01–1.04	0.83	0.82–0.85	0.68	0.66–0.69
*Income level*								
Low (ref)	1		-	-	1		1	
Medium	0.34	0.34–0.35	-	-	0.60	0.59–0.61	0.59	0.58–0.60
High	0.30	0.29–0.30	-	-	0.48	0.48–0.49	0.47	0.46–0.47
Very high	0.21	0.21–0.21	-	-	0.31	0.31–0.31	0.39	0.39–0.40
*Number of visits to primary health care services*								
Number of visits	1.29	1.28–1.29	-	-	-	-	1.27	1.27–1.28
Nagelkerke R Square	-		0.148		0.171		0.332	

Model 1: gender, age and immigrant area of origin; Model 2: Model 1 plus income level; Model 3: Model 2 plus number of visits

**Fig 2:**
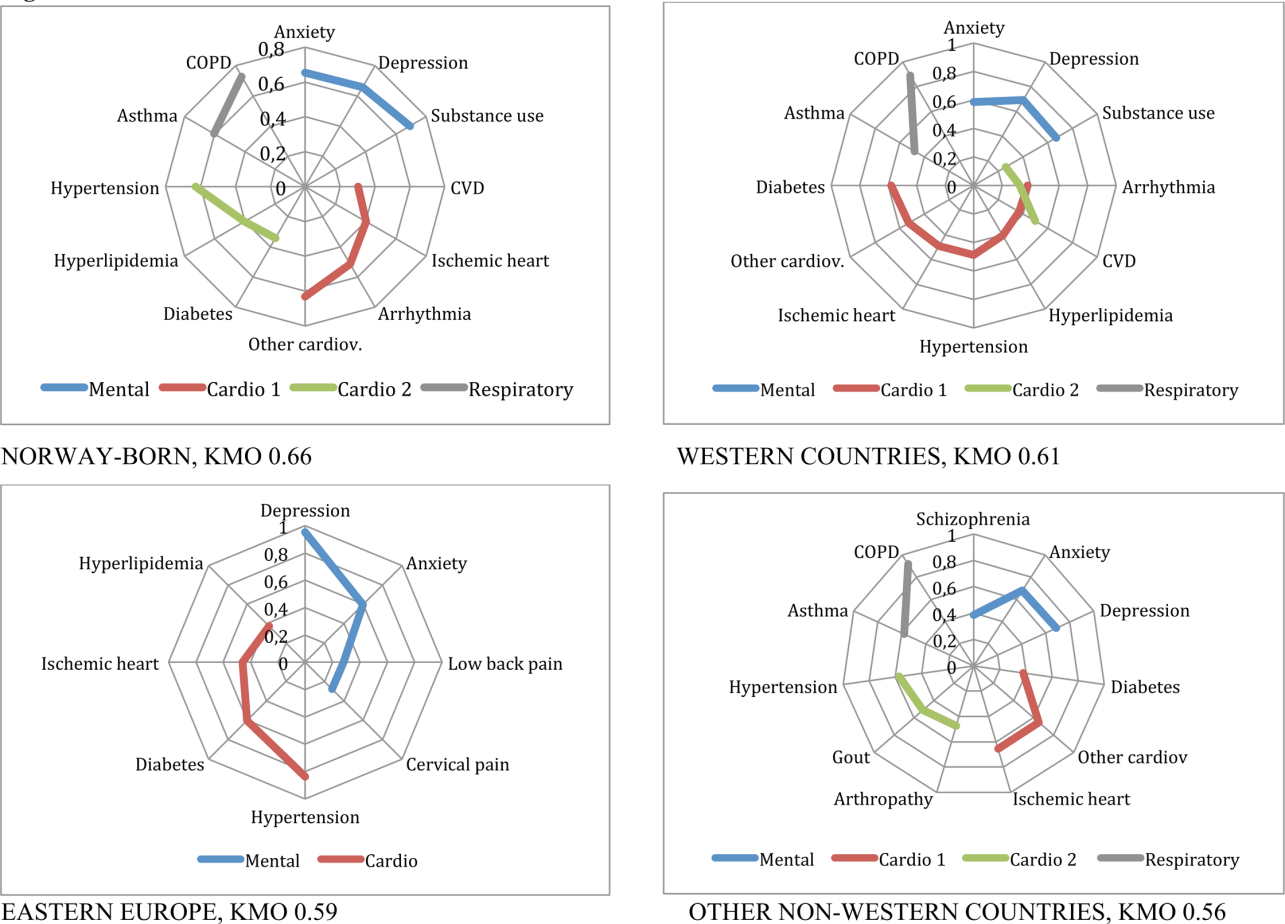
Patterns of multimorbidity among men living in Norway. Men 45 to 64 years old.

**Fig 3:**
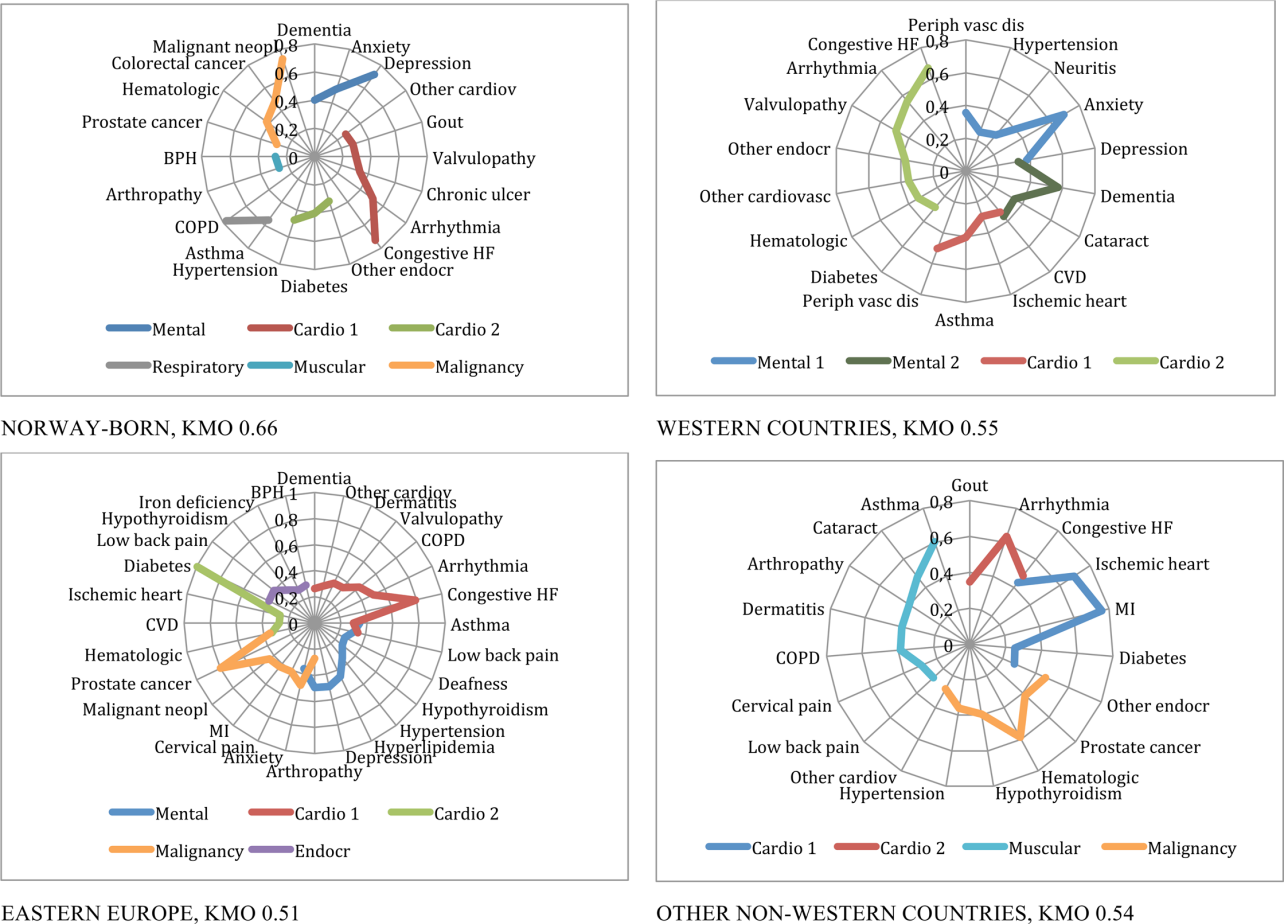
Patterns of multimorbidity among men living in Norway. Men 65 or older.

**Fig 4:**
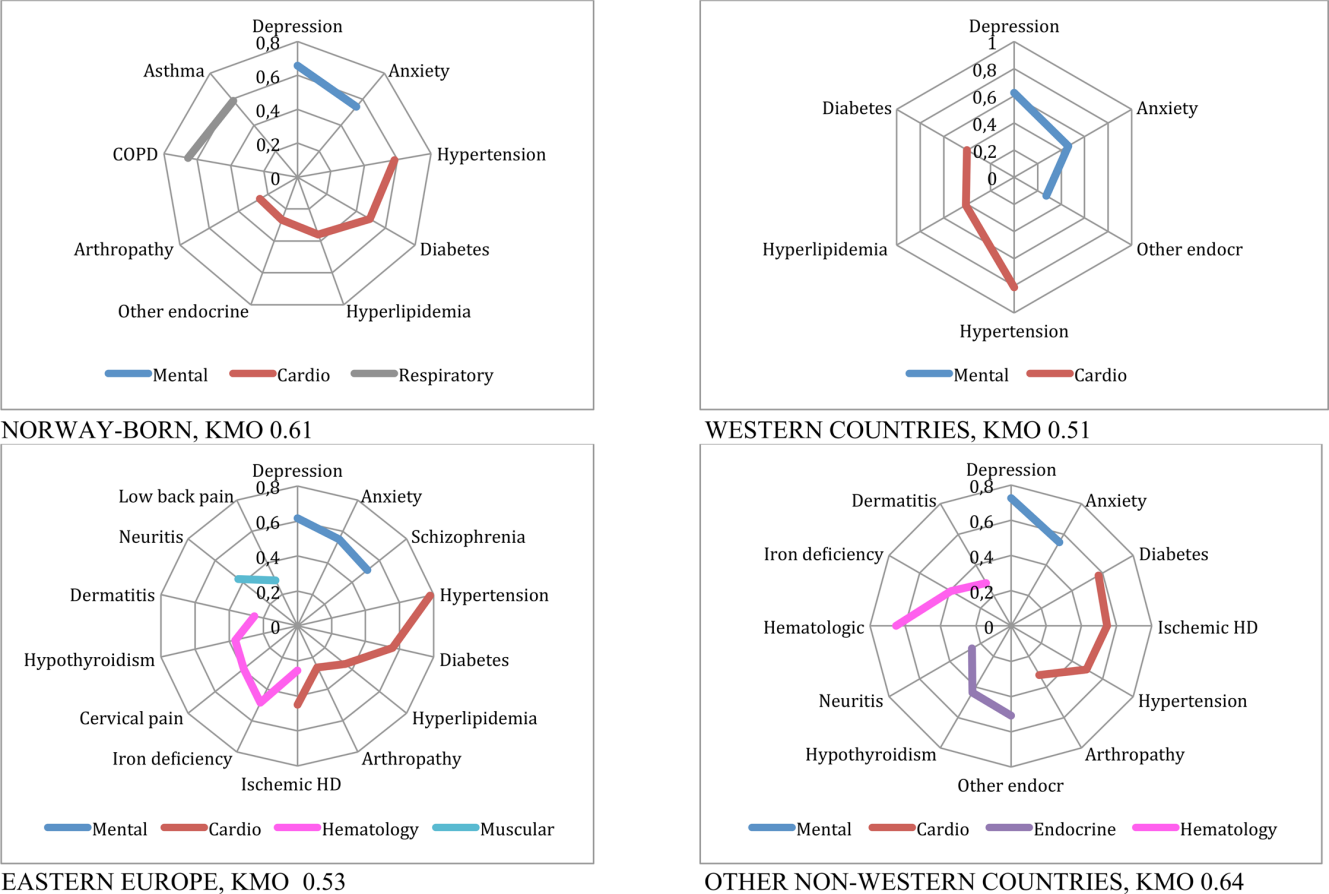
Patterns of multimorbidity among women living in Norway. Women 45 to 64 years.

**Fig 5:**
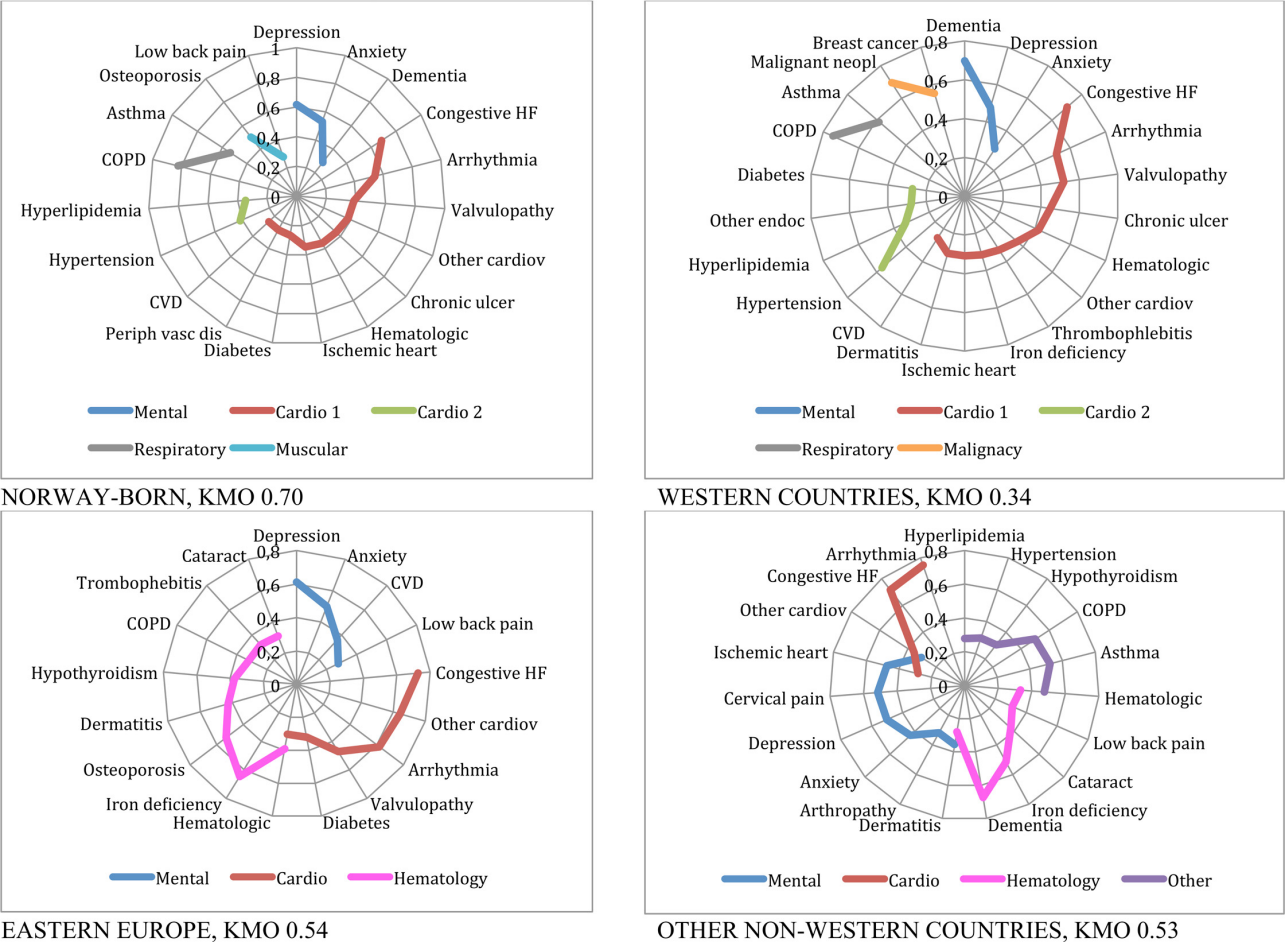
Patterns of multimorbidity among women living in Norway. Women 65 or older.

The multimorbidity patterns for Norwegians and immigrants based on the described factor analyses are presented in Table 3 and Figs 2 to 5, and summarised in Table 4. In the Figures, EDCs belonging to the same pattern are linked through a continuous line in different colours depending on the type of pattern, for example blue for mental or red for cardiovascular patterns. For all groups studied but one, KMO was higher than 05, indicating an acceptable goodness of fit.

**Table 3:** Patterns of multimorbidity and contributing diseases for men and women 15 to 44 years old across groups. Results of factor analyses applying oblique rotation (Oblimin). ^a^

Norwegian-born			Western Countries (West Europe & N. America)			Eastern Europe			Other Non-Western countries (Asia, Africa & Latin America)			
Men 15–44												
*Diseases*	*Mental health*	*Respiratory*	*Diseases*	*Mental health*	*-*	*Diseases*	*Mental health*	*-*	*Diseases*	*Mental health*	*Respiratory*	*-*
	*Score*	*Score*		*Score*			*Score*			*Score*	*Score*	
Depression	0.68	-	Depression	0.55	-	Depression	0.67	-	Depression	0.89	-	-
Anxiety	0.72	-	Anxiety	0.89	-	Anxiety	0.93	-	Anxiety	0.55	-	-
Substance use	0.67	-							Asthma	-	0.29	-
Dermatitis	-	0.36							Cervical pain	-	0.76	-
Asthma	-	0.45										
KMO 0.6776			KMO 0.5451			KMO 0.5317			KMO 0.5929			
Women 15–44												
*Diseases*	*Mental health*	*Endocrine*	*Diseases*	*Mental health*	*Respiratory*	*Diseases*	*Mental health*	*Endocrine*	*Diseases*	*Mental health*	*Endocrine*	*Haematology*
	*Score*	*Score*		*Score*	*Score*		*Score*	*Score*		*Score*	*Score*	*Score*
Depression	0.66	-	Depression	0.70	-	Depression	0.91	-	Depression	0.77	-	-
Anxiety	0.66	-	Anxiety	0.63	-	Anxiety	0.57	-	Anxiety	0.68	-	-
Other endocr.	-	0.58	Dermatitis	-	0.63	Cervical pain	0.34	-	Cervical pain	0.26	-	-
Hypothyroid.	-	0.56	Asthma	-	0.33	Other endocr.	-	0.94	Asthma	0.28	-	-
Hypertension	-	0.29				Hypothyroid	-	0.45	Other endoc.	-	0.42	-
									Hypothyroid	-	0.78	-
									Iron defic.	-	-	0.77
									Hematology	-	-	0.51
KMO 0.637			KMO 0.5327			KMO 0.6118			KMO 0.6704			

^a^ Expanded Diagnostic Clusters with scores equal to or greater than 0.25 for each factor were selected for the relevant multimorbidity patterns.

KMO: Kaiser-Meyer-Olking measure of sampling adequacy

**Table 4:** Expanded Diagnostic Clusters (EDCs*) included in the Patterns of multimorbidity for natives and immigrants by age and gender.

	Norwegian-born		Western countries (West Europe & North America)		Eastern Europe		Other Non-Western (Asia, Africa & Latin America)	
	Patterns	EDCs	Patterns	EDCs	Patterns	EDCs	Patterns	EDCs
Men, 15–44	Mental health	7	Mental health	4	Mental health	3	Mental health	7
	Respiratory/atopic						Respiratory	
Men, 45–64	Mental health	17	Mental health	16	Mental health	9	Mental-psychiatry	15
	Cardiovascular		Cardiovascular		Cardio-endocrine		Cardiovascular	
	Cardio-endocrine		Cardio-endocrine				Cardio-endocrine	
	Respiratory		Respiratory				Respiratory	
Men, 65+	Mental-geriatric	32	Mental health	30	Mental-psychosomatic	28	Cardiovascular	23
	Cardiovascular		Mental-geriatric		Cardiovascular +		Cardio-endocrine	
	Cardio-endocrine		Cardiovascular		Cardio-endocrine		Malignant +	
	Respiratory		Cardio-endocrine		Malignant +		Musculoskeletal +	
	Muscular				Complex endocrine			
	Malignant							
Women, 15–44	Mental health	10	Mental health	6	Mental health	8	Mental health	11
	Endocrine		Respiratory/atopic		Endocrine		Endocrine	
							Haematological	
Women, 45–64	Mental health	17	Mental health	13	Mental-psychiatry	16	Mental health	16
	Cardio-endocrine		Cardio-endocrine		Cardio-endocrine		Cardio-endocrine	
	Respiratory				Musculoskeletal		Endocrine	
					Haematological		Haematological	
Women, 65+	Mental-geriatric	29	Mental-geriatric	29	Mental health	25	Mental-psychosomatic	23
	Cardiovascular		Cardiovascular		Cardio-endocrine		Cardiovascular	
	Cardio-endocrine		Cardio-endocrine		Haematological +		Haematological +	
	Musculoskeletal		Respiratory				Other +	
	Respiratory		Malignant					

*Number of chronic EDCs with a prevalence of 1% or higher included in the analyses.

Men 15 to 44: A *mental health* pattern of depression and anxiety was present in all groups, and included abuse of substances for Norwegians. Both Norway and other non-Western countries showed a *respiratory* pattern of asthma (Table 3).

Men 45 to 64: The *mental health* pattern persisted, and included substance abuse for Norway and Western countries, but was related to pain in Eastern European countries and to schizophrenia in other non-Western countries. Two *cardiovascular* patterns, called Cardio 1 and Cardio 2 in the Figures, one of them including diabetes in a *cardio-endocrine* pattern, emerged for Norway, Western and other non-Western countries. Eastern European countries only showed one *cardio-endocrine* pattern. A *respiratory* (asthma-chronic obstructive pulmonary disease (COPD)) pattern appeared in most groups with the exception of Eastern countries (Fig 2).

Men 65 years and older: Anxiety and depression were associated at this stage to dementia in a *mental-geriatric* pattern for Norwegians and Western patients, who also presented another *mental health* pattern combined with cardiovascular disease. For Eastern Europeans, anxiety and depression were associated to pain as in the younger group, but also to other diagnoses in a more *complex (psychosomatic) mental health* pattern. Depression was not included in any pattern among other non-Western immigrants in this age group. Two *cardiovascular* patterns emerged for all groups, one of them being a *cardio-endocrine* one always including diabetes. The *cardiovascular* pattern included congestive heart failure (CHF), arrhythmia, valvulopathy and other cardiac conditions for most of the groups, but varied widely for the different groups as shown in Fig 3. Another *complex pattern* for Eastern Europeans also included diabetes, iron deficiency, hypothyroidism, low back pain and prostatic hypertrophy. The *respiratory* pattern emerged as a singular pattern for Norway, while asthma and COPD where associated to other patterns for Eastern and other non-Western countries. A pattern of *malignancy* emerged in all but Western countries, with prostate cancer in combination with haematological disease for all groups. These *malignant* patterns were more complex for Eastern Europeans and for other non-Western countries. Last, a *musculoskeletal* pattern containing respiratory disease, cataract and dermatitis appeared in this group (Fig 3).

Women 15 to 44: The *mental health* pattern was also present among young women in all groups, and included cervical pain for Eastern and other non-Western countries and asthma for the latter. With the exception of Western countries, all presented an *endocrine* pattern including hypothyroidism and other endocrine disorders. Western countries presented an *atopic-respiratory* pattern of dermatitis and asthma while other non-Western countries presented a *haematological* pattern of iron deficiency and other haematological disorders (Table 3).

Women 45 to 64: A *mental health* and a *cardiovascular* pattern were common to all countries in this age category. The *mental health* pattern included other endocrine disorders for Western countries and comprised schizophrenia in Eastern European countries. For all groups, hypertension and diabetes were included in the emerging *cardio-endocrine* pattern, and hyperlipidemia in all but other non-Western countries. Arthropathy was also included in this pattern, except for Western countries. Ischemic heart disease was associated to this pattern for Eastern Europeans and other non-Western countries. The *respiratory* pattern appeared only among Norwegians. A *musculoskeletal* pattern combining low-back pain and peripheral neuropathy emerged for Eastern countries. An *endocrine* pattern of hypothyroidism was revealed for non-Western countries. Last, for Eastern Europe and non-Western countries, a *haematological* pattern comprised iron deficiency and dermatitis, in addition to cervical pain, hypothyroidism and ischemic heart disease for the former, and other endocrine disorders for the latter (Fig 4).

Women 65 and older: As for men, a *mental-geriatric* pattern appeared for Norway and Western countries, while depression and anxiety were associated to CVD and pain for Eastern Europeans and to a more *complex (psychosomatic) mental* pattern for non-Western women. Women from Norway and Western countries presented two *cardiovascular* patterns, one of them including diabetes, while only one emerged for East Europeans and other non-Western. The main *cardiovascular* pattern for elderly included CHF, arrhythmia and other cardiac conditions in all groups, and was more complex for Norwegians and women from Western countries compared to Eastern Europeans and non-Western women. However, the *psychosomatic* pattern for this last group was also related to cardiovascular diseases as explained. A second, lighter, *cardiovascular* pattern of hyperlipidemia and hypertension alone appeared for Norwegians. For Western countries these risk factors were associated in a *cardio-endocrine* pattern with diabetes and other endocrine disorders. Among non-Western women, the combination was even more complex. A simple *respiratory* pattern was present for Norwegians and women from Western countries. A *musculoskeletal* pattern was present for Norwegians, and a *malign* one including breast cancer crystallised only for Western Europe. Non-Western and Eastern European women presented complex *haematological* patterns (Fig 5).

## Discussion

Immigration from all areas of origin was in this study negatively associated to multimorbidity. Regarding the existence of multimorbidity patterns, more similarities than differences were observed among Norwegian-born and immigrants, in particular between Norwegian-born and those from Western countries. Although differences were observed in the development of patterns with age, as it was the case with ischemic heart disease among immigrant women, we could not systematically detect the development of the multimorbidity patterns among immigrants at younger ages.

Our results of lower odds of multimorbidity among immigrants align with a recent study reporting OR of 0.1 (0.0–0.8) and 0.8 (0.7–0.9) for immigrants living in Canada under and over 5 years respectively [34] Including approximately 3,7 million persons, our study is, as far as we know, the largest to date identifying patterns of multimorbidity and the first one that includes immigrants. A picture consistent with previous literature emerged, including *cardiovascular-endocrine* patterns comprising a variety of cardio-metabolic conditions, sometimes split into two factors, a *mental health* pattern, and a *musculoskeletal* pattern [1, 2, 9, 11]. There were, however, some noteworthy differences regarding the *mental health* pattern, that was associated to schizophrenia in middle aged men and women from other non-Western and East European countries respectively, in line with the literature in the field [35]. For the elderly, the *mental health* pattern was associated with dementia for Norway, Western countries and women from Eastern European countries. The mental health pattern did not emerge for older men from other non-Western countries and was associated to a more complex *psychosomatic* pattern for older Easter Europeans of both genders. These differences, concordant with existing studies, could reflect the lower proportion of dementia diagnoses among immigrants from low income countries in Norway [36], higher degrees of somatization among immigrants [37] as well as cultural differences, social stigma attached to some diseases, and communication problems for some groups [38].

Three additional patterns of multimorbidity emerged from our data: *malignant*, *haematological*, and *respiratory*. The *malignant* pattern appeared at older ages in all groups and included the most common cancer types [39]. However, this pattern was more complex in East European and other non-Western countries. This findings supports Lyratzopoulus et al in 2012 regarding the intricacy of cancer diagnoses and the higher number of consultations needed to refer immigrant patients with cancer to secondary health care [40]. The *haematological* pattern appeared only in women from Eastern Europe and other non-Western countries. In accordance with the high prevalence of anaemia described among young immigrants [41–43], its main components were iron deficiency and other haematological disorders, but the pattern became more complex with age, especially for Eastern Europeans, with combinations of disease that are hard to explain by classical pathogenesis. These complex patterns might reflect a higher vulnerability to disease as explained by the concept of allostatic overload [44], which has been connected to lower levels of serum erythrocytes and greater mean corpuscular volume [45]. In addition, other explanations include the GPs’ challenges to categorise disease for women from different cultural and linguistic backgrounds [40], and to different presentation of disease among immigrant women compared to Norwegian-born and Western women. Last, the *respiratory* pattern combining COPD and asthma might reflect idiosyncrasies of the Norwegian system for reimbursement of prescriptions. Until recently, there have been no specific pharmacologic treatments of COPD, and the available therapies are “borrowed” from asthma and adapted to COPD [46]. Because of this, some of the COPD treatments could only be reimbursed for patients with a diagnosis of asthma. This, together with difficulties of labelling COPD versus asthma in the clinics is probably the explanation for this consequent pattern.

The strengths of our register study rely on the nationwide coverage, limiting self-selection bias, a common caveat for immigrants, and providing large numbers to enable classification of immigrants in three different groups, despite large heterogeneity within each group. Although we regrouped areas of origin after exploring each of them separately, there is of course variation in disease patterns and prevalences within groups that our study cannot disentangle. Rarely is information on socioeconomic levels available for the entire population, although income level seemed to be less able to differentiate socioeconomic level among the eldest groups. Last, we used both a single count approach and a more sophisticated study of patterns of morbidity, presenting a more complete view of multimorbidity among Norwegian-born and immigrant groups.

The methods for the study of multimorbidity are still evolving [47]. Multimorbidity can be measured by simple counts of diseases in an individual [1], or using indices to assess morbidity burden, that differentially weight a range of conditions, like the ACG System or the Charlson index [5, 48]. Although the most used definition of multimorbidity includes two or more chronic diseases, the cut-off of three chronic diseases has also been suggested as a valid one [10]. Therefore, we conducted analyses for both definitions, but obtained similar results.

Our study was based on diagnoses made by physicians, avoiding self-reported bias, and we subsequently selected the chronic diagnoses included in Salisbury’s list [32] in accordance to previous studies [11, 33]. However, because we used routine data for administrative purposes, our study shares the limitations of other multimorbidity studies, particularly reliance on the quality of the data recorded [49]. Nevertheless, ICPC-2 data from administrative claims is validated for comparison of groups [50, 51], which was our aim. To reduce potential misclassification of diagnoses by the physicians and to increase the comparability of our study with others, we used the EDCs created by the ACG System [5, 52]. Incomplete register of diagnoses is another potential limitation, as the physicians may choose only one diagnosis in a given consultation despite the presence of several diseases. On the other hand, multimorbidity levels of those patients not attending to primary care cannot be registered. However, including the number of visits for each individual to primary health care during the study period in the analyses did not change the direction of the associations between immigrant status and multimorbidity. The “salmon bias” effect, according to which elderly sick patients would travel back to their countries of origin [53], could also have confounded our results, since these patients would not have visited primary care in the study year and thus not been diagnosed. However, recent analyses for other non-Western immigrants in Norway indicate that a low proportion among the elderly move back to their countries of origin [54]. Nevertheless, the low prevalence of multimorbidity among the oldest groups in our study is most likely explained because the HELFO-database does not include consultations for individuals living in nursing homes. Unfortunately, we have no data on the proportion of immigrants that live in nursing homes, but immigrants might be more reluctant than Norwegians to live away from their own homes [55], which would increase the differences in multimorbidity that we find between the Norwegian-born and the immigrant oldest patients. Our figures of multimorbidity are thus lower than the 42% prevalence of multimorbidity reported in a recent study based on self-reported disease in Norway [56] and should not be used as comprehensive prevalences.

Despite the lower levels of multimorbidity among immigrants compared to Norwegians, immigration is often related to lower socioeconomic status, low health literacy [57] and barriers to health care services use [58], which in turn can additionally complicate the impact of multimorbidity on some immigrant groups. In a recent systematic review, general practitioners identified four difficult areas in caring for patients with multimorbidity: disorganisation and fragmentation of care, inadequacy of current disease specific guidelines, challenges in delivering patient centred care, and barriers to shared decision making [2]. Many of these areas are even further complicated when physicians interact with vulnerable immigrant groups [59–62]. Although access to health services is necessary for health care, more access alone does not necessarily result in equitable health care outcomes [63]. Viewing immigrant patients holistically rather than disease-by-disease [8], and increasing the awareness of the complexity of multimorbidity among elderly immigrants might play a major role in developing effective preventive and treatment strategies in the clinical encounter with immigrants.

## Conclusions

Our study confirmed the associations between multimorbidity and immigrant’s area of origin. Immigrants showed a lower prevalence of multimorbidity compared to Norwegian-born, despite the former frequently having lower socio economic and literacy levels. The similarities regarding the type and composition of the multimorbidity patterns found in both groups confirm common physiopathological basis of diseases. The greater complexity of multimorbidity patterns for some immigrant groups requires further investigation. These complexities imply that health care policies and practice will require a more holistic approach for specific population groups in order to meet their health needs and curb and prevent diseases.

## Supporting Information

S1 Table Countries with at least 5% of the individuals within the immigrant group. (DOCX)

## Abbreviations

ACG^®^: Adjusted Clinical Groups
CHF: Congestive Heart Failure
COPD: Chronic Obstructive Pulmonary Disease
CVD: Cardio Vascular Disease
EDC: Expanded Diagnostic Clusters
ER: Emergency Room
GP: General Practitioner
HELFO: Norwegian Health Economics Administration database
ICPC: International Classification of Primary Care
KMO: Kaiser-Meyer-Olkin
NOK: Norwegian Crowns
