# Supplementary material for: Multimorbidity and Its Patterns according to Immigrant Origin. A Nationwide Register-Based Study in Norway
Source: PLoS One. 2015 Dec 18;10(12):e0145233. doi: 10.1371/journal.pone.0145233 (PMC4684298; doi:10.1371/journal.pone.0145233)
Supplement: S1 Table — (DOCX) [file pone.0145233.s001.docx]

S1 Table. Countries with at least 5% of the individuals within the immigrant group

| **Western countries**  **(n=109438)** | | **Eastern Europe**  **(n=99301)** | | **Other non-Western countries (n=181068)** | |
| --- | --- | --- | --- | --- | --- |
| *Country* | *%* | *Country* | *%* | *Country* | *%* |
| Sweden | 24 | Poland | 39 | Iraq | 9,1 |
| Denmark | 16 | Bosnia-Herzegovina | 12,9 | Pakistan | 8,8 |
| United Kingdom | 10,3 | Russia | 10,9 | Somalia | 7,7 |
| Finland | 5,4 | Kosovo | 8,4 | Vietnam | 7 |
|  |  | Lithuania | 6,5 | Iran | 6,8 |
|  |  |  |  | Philippines | 5,7 |
|  |  |  |  | Thailand | 5,2 |
